# Knowledge translation in Iranian universities: need for serious interventions

**DOI:** 10.1186/1478-4505-11-43

**Published:** 2013-11-13

**Authors:** Jaleh Gholami, Sharareh Ahghari, Abbas Motevalian, Vahid Yousefinejad, Ghobad Moradi, Abbasali Keshtkar, Ali Alami, Saeideh Mazloomzadeh, Mohammad Masoud Vakili, Reza Chaman, Bahman Salehi, Omid Fazelzadeh, Reza Majdzadeh

**Affiliations:** 1 Knowledge Utilization Research Center, Tehran University of Medical Sciences, Tehran, Iran; 2 School of Public Health, Tehran University of Medical Sciences, Tehran, Iran; 3 School of Public Health, Iran University of Medical Sciences, Tehran, Iran; 4 Forensic Science Department, Tehran University of Medical Sciences, Tehran, Iran; 5 Kurdistan Research Center for Social Determinants of Health(KRCSDH), Kurdistan University of Medical Sciences, Sanandaj, Iran; 6 Endocrinology and Metabolism Research Institute, Tehran University of Medical, Sciences, Tehran, Iran; 7 Department of Health, School of Public Health; Social Development and Health Promotion Research Center, Gonabad University of Medical Sciences, Gonabad, Iran; 8 Zanjan University of Medical Sciences, Zanjan, Iran; 9 Public Health & Health Education Department, Zanjan University of Medical Sciences, Zanjan, Iran; 10 Shahroud University of Medical Sciences, Shahroud, Iran; 11 Arak University of Medical Sciences, Arak, Iran; 12 Shiraz University of Medical Sciences, Shiraz, Iran

## Abstract

**Background:**

The aim of this study was to assess the status of knowledge translation (KT) in Iranian medical science universities in order to assess the strengths and weaknesses of the most important organizations responsible for producing knowledge in the country.

**Methods:**

The KT activities were assessed qualitatively and quantitatively in nine universities using the Self-Assessment Tool for Research Institutes.

**Results:**

The strengths and weaknesses of universities were determined using seven main themes: priority setting; research quality and timeliness; researchers’ KT capacities; interaction with research users; the facilities and prerequisites of KT; the processes and regulations supporting KT; and promoting and evaluating the use of evidence.

The quantitative and qualitative results showed that the Iranian universities did not have an appropriate context for KT. There were significant shortcomings in supportive regulations, facilities for KT activities, and the level of interaction between the researchers and research users.

**Conclusions:**

The shortcomings in KT were mostly in the area of stewardship and policymaking (macro level), followed by planning and implementation at the universities. In order to strengthen KT in Iran, it should occupy a prominent and focused role in the strategies of the country’s health research system.

## Introduction

The available research is often not implemented and/or leads to ineffective or harmful care in healthcare despite significant investment in research [1]. Nearly all stakeholders involved in health-related decision making, including healthcare providers, patients, managers and policymakers, in developed and developing countries, have faced such challenges [2]. The World Health Organization’s 2004 report *Knowledge for Better Health* advised member states that improving knowledge translation (KT) is one of the main strategies to improve health systems [3].

By definition, KT is “a dynamic and iterative process that includes synthesis, dissemination, exchange and ethically sound application of knowledge to improve the health, provide more effective health services and products, and strengthen the healthcare system” [4]. The translation of knowledge into action is a non-linear and complicated process that takes place in a complex system of interactions between knowledge producers and users [5]. Many factors affect the process in which organizational factors either in the knowledge-producing or -user organizations are of great importance. In research organizations, organizational factors such as policies, strategies, structures and values play critical roles in determining the researchers’ stance towards KT activities [6].

In Iran, forty-three medical science universities work under the supervision of the Iranian Ministry of Health and Medical Education (MoHME). More than 300 research centers established in these universities are considered the main research bodies in the country’s health system. Following integration with the MoHME in 1985, these universities became responsible for providing healthcare at the provincial level, and for educating human resources and conducting research. The MoHME is also responsible for implementing policies and planning in health, medical education and research, for funding and allocating budgets in health research, and for evaluating the research conducted in universities, research centers and institutes [7-9].

Universities and research centers receive research funding from the MoHME, but can also secure research grants from other organizations. Previous studies indicated that only 3–6% of funding for research in the East Mediterranean region countries, including Iran, has come from non-governmental or private sectors [10]. This figure amounted to around 6% in the Tehran University of Medical Sciences (TUMS), which is the biggest medical university in Iran [11]. These findings suggest that the government is the main source of financial support for research in medical universities in Iran.

Following the rapid pace of research in Iran, there is an emerging recognition of the importance of KT by researchers and research managers. Strengthening community-based participatory studies, establishment of a research center focused on KT, conducting training program for researchers and considering criteria on KT in the evaluation of research centers as well as the promotion of researchers are examples of such efforts. Therefore, universities were looking for the ways to improve the KT of their research findings; however, according to previous publications, these measures were not organized or integrated [12].

The present study was designed to assess the status of KT in Iranian medical universities to point out the strengths and weaknesses of the most important organizations responsible for producing health knowledge in the country. This could help health research policy makers determine the kinds of action to be taken in each level of the health research system.

## Methods

### Subjects

Ten out of forty-two Iranian medical universities, affiliated to the MoHME, were selected by stratified random sampling. Iranian medical universities are evaluated annually on the basis of research activities using criteria such as the number of faculty members and researchers, governance and leadership, empowerment, knowledge production and budget, and were ranked using three categories including Type 1 (nine universities), Type 2 (twenty universities) and Type 3 (thirteen universities) [13]. The Type 1 universities are large institutions with a high number of academic staff and high levels of research funds. Those in Type 3, on the other hand, are small universities with fewer academic staff and less research funds; Type 2 universities liein between. For example, at the time of this study the average number of faculty members in Types 1, 2 and 3 were around 750, 120 and 80, respectively [13]. In the present study, three universities were selected from Type 1, three from Type 2, and four from Type 3 universities, using random numbers.

The selected universities were briefed on the objectives and the methodology of the study. Each university was asked to nominate a facilitator responsible for arranging and facilitating the focus group discussions (FGD). S/he was then provided with the data collection tool and the ethical considerations in a written format. Out of ten facilitators, four were vice chancellors and/or research directors. The group also included one vice chancellor for health and four senior researchers.

To include all of the stakeholders’ opinions in the assessment, and to achieve maximum variance in participants, a variety of individuals were invited to attend sessions, including the university’s vice chancellor or the director of research, the members of the research committee, and researchers (at least two faculty members who had published at least three articles relating to applied research). The intention for each focus group was to include at least one professor, one associate professor and one female member, and other stakeholders (one from the healthcare system and another from other organizations such as pharmaceutical companies, the medical equipment industry and/or a public sector domain other than health). When none of these individuals were available – for instance none of the researchers had three articles published in the field of applied sciences – an individual whose criteria was akin to the abovementioned items was invited.

### Measures

Assessment of the KT activities were performed using the Self-Assessment Tool for Research Institutes (SATORI), the validity and reliability of which has been previously evaluated [14]. This tool includes fifty items, each referring to one of the issues which affect KT, and is scored using a five-point Likert scale ranging from 1 (the situation is quite unfavorable and/or there is a dire need for intervention) to 5 (the situation is acceptable and there is no need for intervention).

In the sessions, SATORI items were discussed. The participants were asked to score the items in group. It means that we finally had one score for each item from each FGD session. In each session, discussion took place around each item until a consensus was reached on a definite score. In cases of ambiguity over the meaning of an item, the facilitator was asked to provide the necessary explanation; if this was not satisfactory, s/he was asked to call the principle investigator (PI) for further details.

### Procedures

Two FGDs were held in each of the seven universities, and two universities held one FGD. Each session lasted at least one and a half hours and an average of eight people participated in each session. All sessions were voice-recorded and transcribed verbatim. The transcribed texts, audio tapes and completed questionnaires were then sent to the PI.

### Analysis

One of the ten universities was excluded because the questionnaires and recordings delivered did not meet the necessary level of quality. The data from the other nine universities were then analyzed qualitatively and quantitatively. The transcribed texts were entered in ‘Open Code’ software and analyzed. Initial SATORI domains are based on the stages of production and use of research knowledge [15]. While these initial domains sound rational for the development of the tool, they were not conclusive for finding the weaknesses and strengths of KT in the universities and a new categorization was needed. Therefore, the items were reviewed by two persons independently and were categorized by seven mutually exclusive themes. Then, the qualitative and quantitative analysis was performed using these seven themes including “priority setting, researchers KT capacities, interaction with research users, the facilities and prerequisites of KT, processes and regulations supporting KT, and promoting and evaluating the use of evidence” (the attribution of the tool items to these themes is shown in the Additional file 1). For the quantitative analysis, after initial combination of the items’ scores from the two sessions (if there were two), the mean and standard deviation of each theme for all universities were calculated using its subset items’ score; items were simply weighted the same.

### Ethical considerations

The interviews were recorded after gaining consent from the interviewees. The name of the interviewee and his/her affiliation were omitted while analyzing the data. The research protocol was also approved by the institutional review board of TUMS, which follows the Declaration of Helsinki.

## Results

### Qualitative section

The status of KT was assessed in seven themes and the participants’ quotes are presented below to show their exact impressions.

### Priority setting

In Iranian universities, research priorities were determined by internal university users’ opinions. User organizations’ priorities were taken into account only if they explicitly announced their priorities to the university. Otherwise, the researchers did not make any inquiries regarding their priorities. However, there was cooperation among the stakeholders and general public in determining the research priorities of Community-Based Participatory Research in a few projects.

In most of the universities, there was no defined, accurate and standard mechanism for priority setting in research. As a result, the real research priorities were not identified and research was conducted without taking into account the users’ needs and their active participation.

“Not much budget is dedicated to research and this small amount of money is distributed unevenly and inaccurately.”

“The determined priorities are not real.”

### Research quality and timeliness

The majority of individuals involved in the field of research believed their research quality was acceptable, and the users of their findings trust the research conducted in the universities.

“If they (the external organizations) hadn’t trusted the university, they would not have ordered the project in the first place.”

They added that the time spent by review bodies on reviewing the proposals and on conducting and reporting the results, particularly for the applied projects, were acceptable and the duration of the whole process was satisfactory. Thus, timeliness was one of the strengths identified in most of the studied universities.

“The proposals are reviewed rapidly as a peer-review session is held every week.”

“If a proposal does not contain any significant problem, it will be carried out rapidly.”

In a few universities, the participants claimed that the process is time-consuming and the results are not reported in time.

“The applied projects are not completed in time and the contract deadlines are not met.”

### Researchers’ KT capacities

The researchers were not aware of KT techniques and did not have the required skills in this regard. Only a small number of the researchers had participated in the KT training workshops.

*“Reporting the results of a research to the public and other users is difficult and needs special training*.”

Among the different methods used for KT, the majority of researchers were involved in writing articles, presenting their findings in seminars and at conferences, and transferring knowledge to their students and peers. A small number were engaged in active KT activities, for example through the close participation of potential users in conducting the research and/or sending their findings to the targeted audiences. They did not pay much attention to the users of their findings while preparing the final report of the project.

In seven out of nine universities, the participants questioned the researchers’ role in conveying their research findings to potential users. Even they did not believe that preparing the ground for the application of findings lay in the hands of researchers.

“An individual who is to formulate a health policy should be scientifically capable of reading an article, understanding it, and applying its results.”

“Who performs a research is not responsible to produce a transferrable message.”

### Interaction with research users

Weak interaction between the researchers and users of research findings and the absence of collaborative networks were considered as weak points of KT in many universities.

“We [in our research centers] have great capacities but have not informed any [user] organization as we do not believe it to be necessary.”

In spite of government regulations that mandate governmental executive organizations (that potentially use research results) to collaborate with research organizations, and in spite of government allocating certain parts of its budget to research activities, these organizations did not follow this rule. The majority of researchers spent the university budget on conducting their own projects, and only a few were engaged in collaborative projects with other organizations. In certain universities, the researchers were not even aware of the possibility or process of securing research grants from other organizations.

Collaborative research was mainly conducted among different research groups within the universities, and the stakeholders of such research were rarely engaged in the projects. Active engagement in activities, such as communicating with the media and holding sessions with the decision makers to transfer research findings to its users, are seldom performed.

“The relationship between the university and the users of research results is limited.”

“I believe we don’t even know our target audiences.”

### The facilities and prerequisites of KT

The researchers had access to some of the facilities and prerequisites of KT to strengthen relations with the users. For instance, the research results were mainly available on the university website in the form of an article, abstract and/or report. The organizations could also gain access to information on researchers and their capabilities through these websites. The majority of the universities informed researchers of the research priorities through the same websites.

The researchers considered an insufficient budget as one of the main reasons for the lack of KT activities. Funds were allocated for the dissemination of research results in the form of publishing an article in a scientific journal or attending conferences. This came while the universities did not devote any part of the budget to the other activities of KT as a part of the project. Many research managers, on the other hand, believed that the researchers did not ask for such facilities and did not accurately predict their costs in their proposals.

“If a researcher foresees the cost of KT activities in his proposal and adds the prices to the costs of the proposal, we wouldn’t oppose.”

One of the obstacles named by the researchers of different specialties and from different universities was not having enough time for KT activities. While some of the individuals acted as agents for KT, there was no person or structure in the universities with a defined job description as knowledge broker. At the same time, the researchers failed to benefit from the skills of the KT professionals, and the universities did nothing to overcome their needs.

The required structures for strengthening KT, such as the establishment of Knowledge or Technology Translation Office in the universities, were mainly absent. In two universities they were in their preliminary stages of development. Other existing structures, such as the Office of Communications with Industry, played a marginal role in this respect.

### Processes and regulations supporting KT

Conducting client-oriented research as a means to facilitate the utilization of research results requires regulations for contracting and grants procurement. These were well defined in universities, yet most researchers were not aware of them. Availing of research grants from user organizations depends mainly on the researcher and the university. As a result, more elite individuals and better universities are likely to attract a greater share of budgets and grants.

The intra-university section of the process in Type 1 universities was smoother. In others, however, there were some concerns; the researchers were unfamiliar with the regulations, the process was lengthy, a part of the budget was spent on the universities’ administration, and there was a low tendency to attract grants and use the budgets provided by user organizations. Meanwhile the majority of the universities had ineffective policies for encouraging the uptake of research grants.

The allocation of budget to projects with applicable results, such as systematic reviews or those leading to the development of clinical guidelines was not considered a priority by the universities.

“No one pays attention to the results of a project while approving its proposal.”

The researchers and research managers of the universities believed there were no defined mechanisms or flowcharts for translating the results of a project and considered this one of the main obstacles to performing such activities in the universities.

In the annual evaluation process conducted by the MoHME for ranking research centers and universities, research centers gained credit if and when the results of their projects were used by user organizations. These were also influential in the faculty members’ promotion; yet, many researchers were unaware of evaluation criteria. The KT activities of the researchers were never evaluated and thus the researchers were not motivated to engage in such activities.

“There is not enough motivation for engaging in KT activities, publishing an article is the only stimulus.”

### Promoting and evaluating the use of evidence

The utilization of the results of a project and the effectiveness of these results in decision making were evaluated unsystematically in the form of research projects. These, however, were not performed as part of the KT plan for research. For many researchers, it was of no concern how the results of a project were used.

“No feedback is received and thus no revision or modification is ever made.”

Capacity building for the utilization of evidence was undertaken only for target audiences inside the university, including hospital managers, faculty members as clinical service providers, and lecturers. The universities did not hold capacity building programs for target audiences outside the university.

“Empowerment programs [for the utilization of evidence] are available for the intra-system users but not for the extra-system ones.”

As many of the researchers were members of the university board committee and the decision making committees of the hospitals and the MoHME, the results of their research affected their decisions.

#### Quantitative section

For the quantitative analysis, the seven themes mentioned in the qualitative section were used. In each university, the mean scores for themes were estimated based on the attributes items’ scores (Table 1). Considering the mean score of nine universities, the research quality and timeliness theme received the highest score (2.9), whereas the promoting and evaluating the use of evidence theme received the lowest (1.8).

**Table 1:** Mean scores of the Iran’s medical universities according to themes of the Self-Assessment Tool for Research Institutes (SATORI)

**Theme**	**University mean score**									**Total mean (SD)**
	**1**	**2**	**3**	**4**	**5**	**6**	**7**	**8**	**9**	
Priority setting	2.0	1.8	1.9	2.6	3.4	3.8	1.5	1.6	4.0	2.4 (0.4)
Research quality and timeliness	3.3	2.4	2.9	3.3	3.2	2.4	2.8	2.4	3.6	2.9 (0.6)
Researchers’ KT capacities	2.3	1.1	2.3	2.4	3.0	2.5	2.5	2.4	3.3	2.4 (0.9)
Interaction with research users	2.2	2.1	2.7	2.4	3.1	2.5	1.6	1.9	3.7	2.4 (0.3)
Facilities and prerequisites of KT	2.1	1.5	1.8	1.8	2.2	1.9	2.7	2.2	3.1	2.1 (0.8)
Processes and regulations supporting KT	2.1	1.2	1.9	1.8	2.0	1.3	1.9	1.8	3.3	1.9 (0.6)
Promoting and evaluating the use of evidence	1.8	1.6	2.0	2.0	1.4	1.9	1.1	1.8	3.0	1.8 (0.4)

## Discussion

The KT activities were assessed qualitatively and quantitatively in nine Iranian medical universities. The evaluation was performed using the SATORI and the strengths and weaknesses of the universities were determined using seven main themes.

The findings showed the undesirable status of KT in Iranian medical universities, despite an acceptable research status and an increase in the number of research publications. This is in line with the findings of a previous study conducted in East Mediterranean countries and in TUMS [9,12]. The qualitative results showed that the Iranian universities did not have an appropriate context for KT and there were important shortcomings in supporting regulations, facilities for KT activities and the level of interaction between researchers and research users. The researchers were capable of carrying out quality research in an acceptable time period, but writing an article or presenting the results in conferences were the only means of translating their findings. Most did not have a positive attitude toward KT and were not trained for such activities.

According to the quantitative results and after comparing the mean scores of the themes, they can be classified into three levels: the research quality and timeliness theme gained the highest score; the capacity of human resources (knowledge, skill and performance), priority setting and interactions with research users ranked second; and finally, the lowest ranking was attributed to the facilities and prerequisites of KT, the processes and regulations supporting KT, and the capacity for promoting and evaluating the use of evidence.

Conducting research and producing high quality science, which many researchers consider their responsibility, is showing an increasing trend in Iran. One part of the high score gained in the “research quality and timeliness” theme may be due to the biased scoring of the researchers and the university’s research system. However, the evidence supports the growing trend of research and research products across the country [16].

Setting research priorities in the university, educating and empowering human resources, and collaborating with research users, are the themes which ranked second; these include the resources, strategies and activities planned and performed at the university level, but were not of a desirable status. Meanwhile the facilities and prerequisites needed for KT, and the supportive regulations that require the attention of the administrative bodies of the research system and require interventions at national level, received the lowest score. The low score received by the “promoting and evaluating the use of evidence” theme was also not surprising as the KT activities were not performed well and no promotion or evaluation of the use of evidence was therefore conducted.

Despite the growing focus on KT in many countries, researchers do not consider KT their responsibility. Even in the universities and countries that produce a large share of global knowledge, many researchers do not consider research studies that translate scientific findings into application as a part of their duties or research agenda [6,17]. Therefore, they continue with their traditional passive activities in KT such as publishing articles in peer review journals. Previous studies showed that only a small number of researchers use active techniques for disseminating their research results to audiences [18-20]. This was one of the findings of the present study and highlights that systematic efforts are needed to motivate the researchers to deliver their message to the users and create an environment suitable for KT activities.

According to Jacobson’s findings, even in some universities where researchers are expected to perform mode II or active KT activities and to make an effort to apply their research results practically, the context of academic institutes, including the incentive system, resources and priorities, encourage the researchers towards traditional academic activities. He suggested that in order to strengthen researchers’ KT activities, interventions need to be made at an organizational level in the following areas: promotion and employment rules; budget and resource allocation; modification of structures and shifting the focus towards KT policies; the university’s commitment to KT; teaching students KT skills; employing researchers with the required skills; documentation, standardization and habitualization of KT activities; and planning and promoting the use of evidence [6].

In many developed countries, wide interventions have been undertaken to improve KT. These interventions were not only at university and research institute level, but also at the national level in research funding bodies and healthcare systems [21-24]. Different interventions have been undertaken in the research organizations of these countries, including the development and execution of KT educational programs [25], the provision of guides and toolkits [26,27], and the provision of technical consultancy and supportive services [28]. On the other hand, many health research organizations have established suitable structures for strengthening KT [29,30]. The establishment of the research networks is another attempt at bringing researchers and potential users together and increasing their interaction [31,32]. Awards given for KT, communication and partnership in research, and encouraging researchers towards KT activities are other interventions that have been offered [33].

Requesting collaboration between researchers and knowledge users and the development of a KT plan at the proposal-writing stage is the funders’ strategy to make research more applicable [34]. In research-pioneering countries, KT is a prominent strategy of research organizations and funding bodies, and they strategically plan for enhancing KT [35].

In Iran, following the establishment of the Knowledge Utilization Research Center, which holds educational programs and promotes the tool for evaluating KT activities in universities, some universities have adopted strategies such as establishing KT offices, holding educational programs to promote human resources, and providing incentives to researchers [12]. While the interventions adopted at university and organizational levels can overcome certain obstacles in this regard, it is only through effective, widespread and fundamental changes in the situation of KT that positive results can be produced at the national level.

The self-assessment of universities using the SATORI can draw a real picture of KT in Iranian medical universities. Selecting three universities from each type helped depict the general status of KT in the country. However, interpretation of scores of the quantitative part of the analysis should be viewed with caution as these nine selected university could represent a small sample for the country’s forty-three medical universities. Furthermore, as the tool is used by researchers, administrators, research managers, members of research councils and the users of research in each university, it can clearly highlight the strengths and weaknesses of the organization from the point of view of different stakeholders [14]. On the other hand, the self-assessment of KT activities by researchers and research authorities may cause an overestimation of the items’ scores, especially considering the fact that the person responsible for conducting the sessions were facilitators who mostly had strong ties with the university’s’ research administration. Nevertheless, a comparison of the scores is useful in decision making around intervention areas, considering the qualitative part of this assessment is more important and the quantitative part is complementary.

## Conclusions

There were many shortcomings in different parts of KT in Iran. Despite the measures taken in certain universities to improve the situation, the problems requiring planning and intervention at the macro level are more severe. As a result, the issue should be considered in stewardship and policymaking at the macro level, followed by planning and implementation at the universities. In order to strengthen KT in Iran, it should occupy a more prominent and focused role in the strategies of the country’s health research system.

## Abbreviations

FGD: Focus group discussions; KT: Knowledge translation; MoHME: Ministry of Health and Medical Education; SATORI: Self-Assessment Tool for Research Institutes; TUMS: Tehran University of Medical Sciences.

## Competing interests

The authors declare that they have no competing interests.

## Authors’ contributions

RM gave the idea of this study. JG, SA, AM, VY, GM, AK, AA, SM, MV, RC, BS and OF contributed by data gathering and analysis in different medical universities. JG developed the final format of the analysis. JG and SA compiled data from different source to be harmonized. JG and RM drafted the article and elaborated interpretations. All authors reviewed and approved the final paper.

## Supplementary Material

Additional file 1 Questionnaire items in 7 themes. Click here for file

## References

[B1] Berwick DM Disseminating innovations in health care JAMA 2003 289 15 1969 1975 10.1001/jama.289.15.1969 12697800

[B2] Canadian Institutes of Health Research Evidence in Action, Acting on Evidence 2006 Ottawa: Canadian Institutes of Health Research, Institute of Health Services and Policy Research: A Casebook of Health Services and Policy Research Knowledge Translation Stories 122

[B3] World Health Organization World Report on Knowledge for Better Health Strengthening Health Systems 2004 Geneva: World Health Organization 16 146

[B4] Canadian Institutes of Health Research More About Knowledge Translation at CIHR 2013 Ottawa: Canadian Institutes of Health Research

[B5] Sudsawad P Knowledge Translation: Introduction to Models, Strategies, and Measures 2007 TX: National Center for the Dissemination of Disability Research: Austin

[B6] Jacobson N Butterill D Goering P Organizational factors that influence university-based researchers’ engagement in knowledge transfer activities SciCommun 2004 25 3 246 259

[B7] Marandi SA The integration of medical education and health care services in the irof Iran and its health impacts Iran J Public Health 2009 38 4 12

[B8] Malekafzali H EftekhariMB, PeykariN, Sadat GholamiF, DjalaliniaSh, OwliaP, HabibiE, MesgarpourB, VaseiM: research assessment of Iranian medical universities, an experience from a developing country Iran J Pub Health 2009 38 1 47 49

[B9] Majdzadeh R Nedjat S Denis JL Yazdizadeh B Gholami J: ‘Linking research to action’ in Iran: two decades after integration of the Health Ministry and the medical universities Public Health 2010 124 7 404 411 10.1016/j.puhe.2010.03.026 20537362

[B10] World Health Organization A Study of National Health Research Systems in Selected Countries of the WHO Eastern Mediterranean Region 2004 World Health Organization, Regional Office for the Eastern Mediterranean: Egypt, Islamic Republic of Iran, Morocco, Pakistan and Sudan

[B11] Majdzadeh R Nedjat S Gholami J Nedjat S Maleki K Qorbani M Shokoohi M Ashoorkhani M Research collaboration in Tehran University of Medical Sciences: two decades after integration Health Res Policy Syst 2009 7 1 8 10.1186/1478-4505-7-8 19386131 PMC2679006

[B12] Majdzadeh R NedjatS, FotouhiA, MalekafzaliH: Iran’s approach to knowledge translation Iran J Public Health 2009 38 58 62

[B13] Ministry of Health and Medical Evaluation Monitoring and Evaluation Group: Ranking Resulting from the Evaluation of Research Activities in Iranian Universities and Medical Schools 2010 Teheran: Ministry of Health

[B14] Gholami J Majdzadeh R Nedjat S Nedjat S Maleki K Ashoorkhani M Yazdizadeh B How should we assess knowledge translation in research organizations; designing a knowledge translation self-assessment tool for research institutes (SATORI) Health Res Policy Syst 2011 9 1 10 10.1186/1478-4505-9-10 21342517 PMC3053266

[B15] Majdzadeh R Sadighi J Nejat S Mahani AS Gholami J Knowledge translation for research utilization: design of a knowledge translation model at Tehran University of Medical Sciences J ContinEduc Health Prof 2008 28 4 270 277 10.1002/chp.193 19058259

[B16] MacKenzie D 2010 Iran Showing Fastest Scientific Growth of any Country [http://www.newscientist.com/article/dn18546-iran-showing-fastest-scientific-growth-of-any-country.html]

[B17] Weston CM Bass EB Ford DE Segal JB Faculty involvement in translational research and interdisciplinary collaboration at a US academic medical center J Investig Med 2010 58 6 770 776 20517162 10.231/JIM.0b013e3181e70a78

[B18] Lavis J Robertson D Woodside JM McLeod CB Abelson J Knowledge Transfer Study Group: How can research organizations more effectively transfer research knowledge to decision makers? Milbank Q 2003 81 2 221 248 10.1111/1468-0009.t01-1-00052 12841049 PMC2690219

[B19] Sixth Annual Survey of Knowledge Transfer Activities in Public Sector Research Establishments (PSREs) Brighton 2011 Department for Business Innovation and Skills

[B20] Nedjat S Majdzadeh R Gholami J Nedjat S Maleki K Qorbani M Shokoohi M Ashoorkhani M Knowledge transfer in Tehran University of Medical Sciences: an academic example of a developing country Implement Sci 2008 3 1 39 10.1186/1748-5908-3-39 18727835 PMC2538542

[B21] Canadian Institutes of Health Research Innovation in Action: Knowledge Translation Strategy 2004–2009 2004 Canadian Institutes of Health Research: Ottawa 14

[B22] Zerhouni E The NIH Roadmap Science 2003 302 5642 63 72 10.1126/science.1091867 14526066

[B23] Improving Knowledge Transfer Between Research Institutions and Industry Across Europe Improving Knowledge Transfer Between Research Institutions and Industry Across Europe 2007 European Commission: Brussels

[B24] House of Commons Science and Technology Committee; Willis P Research Council Support for Knowledge Transfer 2006 Fifth Special Report of Session 2005–06. London: Stationery Office: Government Response to the Committee’s Third Report of Session 2005–06 13

[B25] Kho ME Estey EA DeForge RT Mak L Bell BL Riding the knowledge translation roundabout: lessons learned from the Canadian Institutes of Health Research Summer Institute in knowledge translation Implement Sci 2009 4 33 10.1186/1748-5908-4-33 19523216 PMC2700786

[B26] Fernández-Peña JR Moore L Goldstein E Decarlo P Grinstead O Hunt C Bao D Wilson H Making sure research is used: community-generated recommendations for disseminating research ProgCommun Health Partnersh 2008 2 2 171 176 10.1353/cpr.0.0013 20208251

[B27] Toolkit for researchers International Development Research Centre 2013 [http://www.idrc.ca/EN/Resources/Tools_and_Training/Pages/Toolkit-for-researchers.aspx]

[B28] Specialized Technical Assistance Services for NIDRR Grantees National Center for the Dissemination of Disability Research 2013 [http://www.ncddr.org/]

[B29] Van Olphen J Ottoson J Green L Barlow J Koblick K Hiatt R Evaluation of a partnership approach to translating research on breast cancer and the environment ProgCommun Health Partnersh 2009 3 3 213 226 10.1353/cpr.0.0081 PMC2836491 20208222

[B30] Technology Commercialization and Knowledge Transfer Office Columbus 2013 OH: Ohio State University

[B31] Conklin J Stolee P A model for evaluating knowledge exchange in a network context. [Erratum appears in Can J Nurs Res. 2008 Sep;40(3):preceding table of contents] Can J Nurs Res 2008 40 2 116 124 18714901

[B32] Curtis McMillen J Lenze SL Hawley KM Osborne VA Revisiting practice-based research networks as a platform for mental health services research Adm Policy Ment Health 2009 36 5 308 321 10.1007/s10488-009-0222-2 19399606 PMC3755587

[B33] Knowledge Translation CIHR Newsletter e Ottawa: Canadian Institutes of Health Research 2012 2013

[B34] Ridde V Knowledge transfer and the university system’s functioning: need for change. Glob Health Promot 2009 16 3 3 5 10.1177/1757975909339768 19773295

[B35] Health Research Roadmap Creating Innovative Research for better health and Health Care 2009 Ottawa: Canadian Institutes of Health Research: CIHR’s Strategic Plan [http://www.cihr-irsc.gc.ca/e/40490.html]

