# Supplementary material for: Knowledge translation in Iranian universities: need for serious interventions
Source: Health Res Policy Syst. 2013 Nov 13;11:43. doi: 10.1186/1478-4505-11-43 (PMC3835863; doi:10.1186/1478-4505-11-43)
Supplement: Additional file 1 — Questionnaire items in 7 themes. [file 1478-4505-11-43-S1.doc]

Additional file 1

Table 1: Questionnaire items in 7 themes

| Theme | **^†^Questionnaire items** |
| --- | --- |
| Priority setting1 | -3, 1-6, 1-7, 1-8 |
| Research quality & timeliness | 2-3, 2-4, 2-5, 2-6, 2-7, 2-8, 3-16 |
| Researchers' knowledge translation capacities | 3-3, 3-4, 3-5, 3-11, 3-12, 3-20 |
| Interaction with research users | 1-1, 1-2, 1-4, 1-5, 2-2, 3-19 |
| Facilities and prerequisites of KT | 1-9, 2-9, 3-6, 3-7, 3-8, 3-9, 3-13, 3-14, 3-18 |
| processes and regulations supporting knowledge translation | 1-10, 1-11, 1-12, 2-1, 3-1, 3-2, 3-10, 3-15, 3-17, 3-21, 3-25 |
| promoting and evaluating the use of evidence | 3-22, 3-23, 3-24, 4-1, 4-2, 4-3, 4-4 |

^*^The items of the SATORI tool are present in the paper as an attachment that is openly accessible; (Gholami J, Majdzadeh R, Nedjat S, Nedjat S, Maleki K, Ashoorkhani M, Yazdizadeh B: How should we assess knowledge translation in research organizations; designing a knowledge translation self-assessment tool for research institutes (SATORI). Health Research Policy and Systems 2011, 9(1):10.) http://www.health-policy-systems.com/content/9/1/10
